# Motion Tracker: Camera-Based Monitoring of Bodily Movements Using Motion Silhouettes

**DOI:** 10.1371/journal.pone.0130293

**Published:** 2015-06-18

**Authors:** Jacqueline Kory Westlund, Sidney K. D’Mello, Andrew M. Olney

**Affiliations:** 1 MIT Media Lab, Cambridge, Massachusetts, United States of America; 2 Department of Psychology & Department of Computer Science, University of Notre Dame, Notre Dame, Indiana, United States of America; 3 Department of Psychology, University of Memphis, Memphis, Tennessee, United States of America; University of Akron, UNITED STATES

## Abstract

Researchers in the cognitive and affective sciences investigate how thoughts and feelings are reflected in the bodily response systems including peripheral physiology, facial features, and body movements. One specific question along this line of research is how cognition and affect are manifested in the dynamics of general body movements. Progress in this area can be accelerated by inexpensive, non-intrusive, portable, scalable, and easy to calibrate movement tracking systems. Towards this end, this paper presents and validates Motion Tracker, a simple yet effective software program that uses established computer vision techniques to estimate the amount a person moves from a video of the person engaged in a task (available for download from http://jakory.com/motion-tracker/). The system works with any commercially available camera and with existing videos, thereby affording inexpensive, non-intrusive, and potentially portable and scalable estimation of body movement. Strong between-subject correlations were obtained between Motion Tracker’s estimates of movement and body movements recorded from the seat (*r *=.720) and back (*r *= .695 for participants with higher back movement) of a chair affixed with pressure-sensors while completing a 32-minute computerized task (Study 1). Within-subject cross-correlations were also strong for both the seat (*r *=.606) and back (*r *= .507). In Study 2, between-subject correlations between Motion Tracker’s movement estimates and movements recorded from an accelerometer worn on the wrist were also strong (*r*s = .801, .679, and .681) while people performed three brief actions (e.g., waving). Finally, in Study 3 the within-subject cross-correlation was high (*r* = .855) when Motion Tracker’s estimates were correlated with the movement of a person’s head as tracked with a Kinect while the person was seated at a desk (Study 3). Best-practice recommendations, limitations, and planned extensions of the system are discussed.

## Introduction

The last decade has brought forth a renewed interest in understanding the complex yet intricate relationship between the mind and body. For example, postural control and postural sway have been shown to be affected by working memory load [1,2] and attentional demands [3,4]. Posture and body movement have been shown to convey emotional intensity as well as emotion-specific information [5] and can influence perceptions of facial displays of emotion [6–8]. Posture and general body movements have also been implicated in studies of behavioral mirroring during social interaction [9], in spontaneous interpersonal postural coordination [10], in studies of attention and sensory integration [11,12], and during experiences of cognitive disequilibrium [13].

Simply put, the body and mind are intricately connected. As such, there is a pressing need to be able to accurately monitor body posture and body movements over time and in a wide variety of contexts. Body monitoring systems are needed in both the laboratory and real-world settings. They should be able to accurately track both subtle (e.g., aspects of gait) as well as more pronounced movements (e.g., aspects of postural sway, specific gestures). They should be usable for different populations (e.g., children, adults, elderly, disabled) and in tasks that range from a few minutes to several hours. Developing systems that satisfy these wide-ranging constraints is not easy. As a result, many systems have been developed, each targeting different needs based on the requirements of different research agendas. These systems can be classified as (a) contact sensors, (b) non-contact sensors, (c) mixed systems, or (d) manual approaches, and are briefly discussed below. These systems can be classified in many other ways as well (e.g., by the type of energy involved, whether the system or sensor is physically in contact with the person), so our classification is merely a practical one for the sake of discussion.

### Contact sensors

Contact sensors span two general types. First, there are body-worn accelerometers, magnetometers, and gyroscopes (e.g., [9,14,15]; and see [16] for a review of accelerometry-based motion detectors). As an example, Feese et al. [9] placed a set of six inertial measurement units on the arms, back, and head to measure posture mirroring during a social interaction task. They used data collected during the task to build an automated classifier for three simple arm positions (left arm up, right arm up, both arms up). Since inertial sensors can drift out of calibration, they are often mounted very closely to the body in skin-tight garments. Sensor-integrated garments can be quite effective, though their effectiveness is often related to how well the garments fit the user (e.g., see [15,17]), thereby requiring customized garments to be available if multiple individuals are to be monitored. They are not affected by illumination changes (like some vision-based approaches), and can be used to measure specific limbs or areas of the body. Some potential drawbacks include the expense, the social and physical discomfort of wearing the sensors, and high power consumption (e.g., [17]), though efforts have been made to reduce the cost and power consumption of sensor-integrated garments (e.g., [15,18]).

The second type of contact sensors includes garments made with elongation-sensitive yarns, conductive elastometers, or other bend sensors (e.g., [18–20]). Tognetti et al. [20], for example, integrated conductive elastomer sensors into a fabric glove and into the arm and shoulder of a shirt. They used these to develop software that could record a set of defined postures, recognize recorded postures, and playback postures using 3D animations. These kinds of garments tend to be skin-tight because they rely on the body deforming or straining the sensors in the fabric during movements, which may affect social and physical comfort. One effort to increase physical comfort is via use of plastic optical fiber sensors (e.g., [21]; also see [22], for a review of further fiber optic sensors as well as many other devices for measuring spinal posture).

### Non-contact sensors

Non-contact systems are primarily camera-based. Camera-based systems capture video of individuals for subsequent movement analysis (e.g., [23–25]). Recently, the Microsoft Kinect [26] has been used for near real-time full-body motion capture and gesture recognition (e.g., [27–29]). The Kinect provides sensor data from a color camera, an infrared projector and camera that uses a structured light approach to calculate depth, and a microphone array. The depth camera has proven especially useful. For example, it was used by Biswas and Basu [28] to train a classifier to recognize a set of eight human gestures. In a similar vein, Burba et al. [29] used the depth camera to sense subtle nonverbal behaviors such as respiratory rate and fidgeting behavior. As these examples suggest, the Kinect does offer a viable solution for body movement tracking. However, although the Kinect sensor is a mass-market device with a relatively low price point, it shares many of the features of commercial systems with specialized hardware. First, it cannot be used to analyze existing video—data must be collected using the sensor. Second, specific system requirements must be met to use the sensor, including CPU, RAM, and video card requirements. For some research agendas, the fact that other sensors can be smaller, more discrete, and less distracting than a Kinect is worth noting. Finally, the most relevant concern is that the Kinect is not necessarily easy to use—e.g., free software for skeleton tracking exists, but it is not a “plug and play” solution.

### Mixed systems

Mixed systems used a combination of contact and non-contact sensors, such as high-speed kinematic systems, and pressure or force sensors. For example, several commercially available kinematic systems are available (e.g., Vicon Motion Capture [30]; Optotrak Certus [31]). These products use high-speed cameras (non-contact) to track markers placed on objects or people (contact). They operate in three dimensions, and tend to have high resolution and high accuracy. These are often a good choice if one has the budget. Mixed systems are sophisticated and require careful setup and calibration to realize their potential.

Other mixed systems use pressure or force sensors placed on the seat and back of a chair (see, e.g., [32–34]). Some commercial pressure-sensor arrays are available, such as the Body Pressure Measurement System (BPMS) from Tekscan [35] and other standard force plates (e.g., from AMTI [36]). To compensate for the expense of such systems, lower-cost (and lower-fidelity) alternatives have been devised. For example, Olney and D'Mello [34] constructed a system with two Wii Fit game controller boards that yield 8 pressure data streams, as opposed to the 38×41 sensing array of the Tekscan BPMS. The Wii Fit boards communicated wirelessly with a computer via a Bluetooth connection. However, depending on the Bluetooth stack employed by the host computer, calibration could be difficult or impossible. Custom soldering was also required to address power consumption issues. Arroyo et al. [32] also used low-cost/low-resolution pressure-sensitive pads for a chair, but noted that some real-life practical problems limited use of the sensor to about half of their data collection period. In cases where resolution is still high, such as in the pressure-sensing array built by Kamiya et al. [33] with 64 Tekscan Flexiforce sensors, cost is also higher, and challenges still remain in constructing a reliable, effective system.

### Manual approaches

Most manual approaches consist of trained human coders observing individuals' body movement and making judgments about what movements were made. This can be done in real-time, but more often, videos of individuals' movement are recorded for later analysis. For example, Friesen, Ekman, and Wallbot [37] trained judges to classify hand movements from videotapes of conversations into three categories: speech illustrators, body manipulators, or actions conveying symbolic information. Other coding schemes include the Body Action and Posture Coding System (BAP) [38] for classifying body movements and postures, and the Davis nonverbal state and nonverbal communication scales [39,40] for coding nonverbal aspects of communication and movement behavior, among others (e.g., [41–43]). In these systems, high inter-coder reliability is generally demonstrated, but the coding process can be labor intensive in terms of human capital, as video is sometimes coded frame-by-frame to ensure accuracy (e.g., [42]).

### Challenges and Proposed Solution

Although the different approaches described above provide viable solutions towards monitoring body posture and movement, every solution has its own advantages and disadvantages. These pros and cons must be weighed by each researcher when deciding which system to use, based on the requirements of their particular research questions. Contact sensors and mixed systems provide precise measurements at high temporal resolutions, but can be expensive, intrusive, and might require sophisticated hardware and software. Vision-based non-contact systems, address some of these challenges, but are generally dependent on good lighting conditions and camera positioning. Manual-coding methods are technologically cheap and non-intrusive but can be labor-intensive, and subjective. Even in cases where a one-size-fits-all wearable sensor garment is available [21] or a pressure-sensor can be fabricated at a relatively low cost [32,34], other factors such as limited portability, computational power required, low precision, lack of scalability, special training needed to appropriately use the system, or sensor damage may limit their feasibility in some circumstances, and must be weighed against the benefits to the specific research questions under study.

We propose a vision-based alternative to the available approaches to automatically monitor *body movements*. Specifically, we use robust established background subtraction and motion tracking algorithms to track individuals’ bodily movements over time from a video of the individual’s body while engaging in a task. The algorithms have been previously developed and applied for tracking of simple posed gestures and are well-known for their computational simplicity and robustness [44,45]. Video for the present system can be recorded using any camera; hence, data collection with our software is largely inexpensive and non-intrusive. No other hardware is required for data collection, and because many laptops and tablets have integrated cameras, even the purchase of an external camera may be unnecessary. Furthermore, the software can also be used to analyze huge corpora of existing videos, thereby affording researchers the ability to investigate a previously unexplored data stream and has the capability to run in batch mode for this purpose. Therefore, the proposed solution may be especially helpful for researchers in small or low-budget labs; researchers who want a plug-and-play system or who have minimal programming experience; or for research where wearable instrumentation may bias results.

At this time, the present approach provides a single index of general body movement. This is an important indicator of cognitive and affective processing for a number of reasons. First, bodily movements are expected to be related to arousal, which is a critical component of all major theories of emotion (e.g., see [46–51]). Second, one key function of emotions is to activate action systems, which involve the body, so tracking movement provides insight into how emotions motivate the organism to act [52]. Third, general bodily movements can also signal different nonverbal behaviors, such as fidgeting (generally more movement) or concentration (generally less movement). Fourth, the amount of body movement is a key component of several manual coding systems. For example, BAP uses the extent of articulation of a body or posture action and the temporal length of the action to determine how subtle or pronounced it is [38]. The proposed system offers an automated way to compute this measure. Fifth, recent work has shown that the low-level dynamics of body movement can reveal states of cognitive distress, such as anxiety, confusion, and frustration [13,53], so there is much to be gained from reliably monitoring a single index of bodily movement. Finally, this index of body movement comprises both high-frequency and low-frequency movements. Researchers interested in one or the other can simply apply available digital filters to the movement time series.

It should be noted that the idea of using computer vision techniques to track movement in video is not new (for surveys and reviews, see [54–57]). However, many of the existent video-based human posture and movement analysis systems use a variety of computationally expensive computer vision techniques to classify video segments into particular movement or posture categories. A common approach is to first identify postural features, body shapes, or movement sequences, and then match these features, shapes, and sequences with predetermined categories of movement or posture (e.g., [25,28,58,59]. Several of these systems have been designed for surveillance applications (e.g., [25]) As such, they focus on general human recognition, and larger-scale posture and movement categories (i.e., walking, bending, crawling) rather than on the much smaller changes in posture and movement that are of interest to the cognitive, affective, and social sciences (e.g., [1,3,4,10–13]). Pedestrian tracking, which focuses on detecting and classifying objects as human pedestrians in surveillance and advanced robotics applications, is a special case of general human motion tracking [60]. However, shape and texture are often used for detection, so motion through time may not necessarily be considered. Although the present approach also uses computer vision techniques to estimate movement from video, it avoids computationally expensive algorithms and complicated face, body, or limb models, provides near-real time performance, and is able to detect both very large and very small changes in movement over time.

Other related work includes frame-differencing methods and motion energy analysis techniques from clinical studies [61,62] and experimental psychology [63,64], as well as optical flow techniques from computer science [65,66]. These methods track changes in pixel values frame-by-frame, which, when the background is assumed static, indicate the motion of the object of interest. Paxton and Dale [64], for example, presented a MATLAB script that uses a frame-differencing method to analyze the body movement of pairs of seated people, which they used to measure interpersonal synchrony. They validated their software against hand-coding methods. Similarly, Ramseyer and Tschacher [62] used motion energy analysis to determine nonverbal synchrony between patients and therapists. First, they hand-selected regions of interest in the video that contained the upper body. The background was assumed to be static. Then they computed differences in grayscale pixels across consecutive video frames at 10 frames per second. We also use frame differencing methods. However, a key difference of our method from that of [62] is that we do not assume a completely static background—instead, we include a binary threshold that helps capture just foreground motion while filtering out background noise. Such threshold filters have been used by other researchers (e.g., [61,64,65]); and indeed, many optical flow techniques include filters [65]. We should note that while this filter helps remove background noise, our method does still work best with a largely static background as well. Because our method is continuously updating a model of background, it does not need to be calibrated to a specific background. It is also not using the texture, color, or edges of the background. Thus it is robust to backgrounds with varied texture, color, and edges, and it is relatively robust to backgrounds that change, as long as the magnitude of the change is well below the magnitude of motion that we want to detect. Slow changes in illumination, such as the sun going behind the clouds, or slow changes in motion, such as the sun’s shadow moving across the wall, are both examples of change that are sufficiently slow as to introduce negligible noise.

The present approach is similar to the Eyesweb Motion Analysis Library’s “quantity of motion” measure [23]. This measure was determined by first detecting full body silhouettes in each video frame, then creating silhouette motion images that captured information about differences in silhouette shape and position over the last several video frames. In the present approach, we used motion history images, which are similar to the silhouette motion images used by [23]. However, their algorithm differed from a motion-history image in that it did not include the most recent frame in the history, thus considering only past motion, not the current position of the body. In addition, the algorithm required video of the full body, and was primarily used to analyze full-body gestures and motion during dance.

The present approach is also related to earlier approaches used to monitor the activity of livestock (e.g., [67,68]). However, tracking movement in livestock is not equivalent to tracking human motion, though some similarities do exist. Kristensen et al. [68] used a "scene camera" viewing the livestock from above, which meant the three-dimensional movements were essentially compressed into a two dimensional plane. While this may be sufficient for tracking livestock, which are not bipedal nor have arboreal ancestry and thus have fewer degrees of freedom in how they interact with the world, the question of whether this setup will work equally well for tracking human motion remains an empirical question. A scene camera with a view from above may be sufficient to track humans moving through space, but it may not capture the dimensions of human motion most relevant to studying nonverbal behavior or the dynamics of subtle body motion. As such, the current work targets different dimensions (face-on instead of from above), and could be considered a new application of these livestock tracking techniques in an entirely new domain, with new challenges. The proposed system is a different implementation and validation of a simple way to track human motion for experimental studies of nonverbal behavior.

### Validation Studies

Our primary contribution is to provide a validated, streamlined package for general human motion estimation that is built on elements of established computer vision techniques as described above. We validate our approach by correlating the system’s estimated movement with the output of three different commercial measurement systems. Furthermore, we provide software with a graphical user interface (GUI) in order to make it easy for researchers to collect and analyze motion from video.

In order to validate the present approach, we analyzed data from three sources: (1) an existing study that recorded videos of 28 participants while they engaged in a computerized task [69], (2) the University of Texas at Dallas Multimodal Action Dataset (UTD-MAD) [70], which contains recordings of 8 people performing different actions, and (3) video and Kinect data of a person seated at a desk. In all three validation studies, estimated general body movements were calculated by analyzing the videos with our Motion Tracker software. These estimated body movements of participants were correlated with movements that were simultaneously recorded with another sensor. In the first validation study, this sensor was a commercially-available body pressure tracking system—the Body Pressure Measurement System (BPMS) from Tekscan [35]; in the second validation study, the sensor was a three-axis accelerometer; in the third study, the sensor was a Kinect tracking the participant’s head pose. We expected that the magnitude of the estimated movements from the present approach would strongly correlate with movements recorded from BPMS, the accelerometer, and the Kinect. If so, this approach could be used as a viable alternative for monitoring general body movement.

Although both the BPMS and the accelerometer are contact-based, and the present approach is vision-based, the use of these sensors for validation is justified because our goal was to develop a single measure of general body movement, not a detailed kinematic model of motion like with sophisticated kinematic systems (e.g., Vicon Motion Capture [30]; Optotrak Certus [31]). In the BPMS, pressure and force sensors capture movement by measuring the ground reaction forces generated by movement, thus providing a measure of general body movement. Importantly, prior work has shown that human motion can largely be reconstructed from ground reaction forces [71]. In addition, other researchers have already used pressure sensors to successfully track and classify motion, though these researchers’ goals differ from our own in what they were primarily studying (e.g., [72–74]. Slivovsky and Tan [73], for example, used pressure sensors to track posture while seated with high accuracy. One aspect of motion we are interested in capturing is small changes in posture, which would easily be captured by pressure sensors placed on the seat and back of a person's chair. Harada et al. [72] measured gross body movement as well as slight movements of a bed-ridden human from pressure sensors placed in the bed, though they did ask participants to minimize movement, rather than instructing participants to move freely or to perform specific body actions. Watanabe et al. [74] also measured body movements while sleeping with a flat pressure sensor placed in the subject's bed, though they focused on measuring biometric signals and did not ultimately find body movement to be the most useful metric. Because much of this prior work with pressure-based contact sensors had different research goals than our own work, we had concerns that these prior studies could sufficiently justify use of the BPMS in measuring movement. As such, we performed a second validation study with data from a three-axis accelerometer worn on the wrist. As discussed earlier, there is a rich history of using accelerometers in contact-based systems to monitor motion (e.g., [9,14–16]).

There are other advantages to using contact-based sensors to validate our vision-based system compared to other vision-based systems. Importantly, a vision-based approach requires more estimation and is more likely to be affected by other artifacts than a contact-based system such as the BPMS or the accelerometer (though note that the Kinect has been successfully validated against Vicon systems, e.g., [75]). Thus, since the present approach is a vision-based system, we were interested in validating it against contact-based systems to rule out the possibility that the correlations might simply be attributable to artifacts, such as changes in lighting and occlusions. However, in order to address a few concerns raised during the first study regarding the alignment of the data streams, in our third validation study, we compared our system to the Kinect’s head-tracking system.

## Overview of the Motion Tracking System

### The Algorithm

In broad terms, the motion tracking algorithm steps frame-by-frame through the video and computes the amount of motion in each frame *F*_*t*_ by measuring the proportion of pixels in *F*_*t*_ that have been displaced (i.e., motion is greater than a predefined threshold) from a moving background model constructed on the basis of *N* earlier frames ([44,45]; for a survey of MHI methods, see [76]). The proportion of “moving” pixels provides an index of the amount of movement in each frame. For example, say *F*_*t*_ is a video frame (image) with 100 (10 × 10) pixels. The background model indicates which of these pixels in the earlier frames (*F*_*t-1*_, *F*_*t-2*_, and so on) have changed from frame to frame in the past. By comparing *F*_*t*_ to this background model, we can tell which pixels have "moved"—that is, what has changed in *F*_*t*_ relative to the earlier frames. If 10 pixels have "moved", then the index of movement is 10/100 = 0.1. Absolute change in the amount of movement in adjacent frames provides an index into changes in movement over time and is taken to be the primary dependent measure. For example, if the proportion of "moving" pixels in frame *F*_*t*_ is 0.1 and in frame *F*_*t+1*_ is 0.3, then the change in movement (or motion) from *F*_*t*_ to *F*_*t+1*_ is 0.2.

The background model is critical for accurate motion filtering. The basis of the model is a motion history image (MHI), an image in which intensity of each pixel is represented as a function of how recently motion has occurred at that pixel over a sequence of images (i.e., the history or memory) [44,45]. Brighter values correspond to more recent motion. The image sequence is the MHI's "memory." At each step of the algorithm, the latest video frame *F*_*t*_ is processed (processing steps are below). This processed image is added to the MHI at maximal brightness. At the same time, all the older images in the MHI memory are decreased in brightness, so that older motion is shown with less intensity.

**Fig 1:**
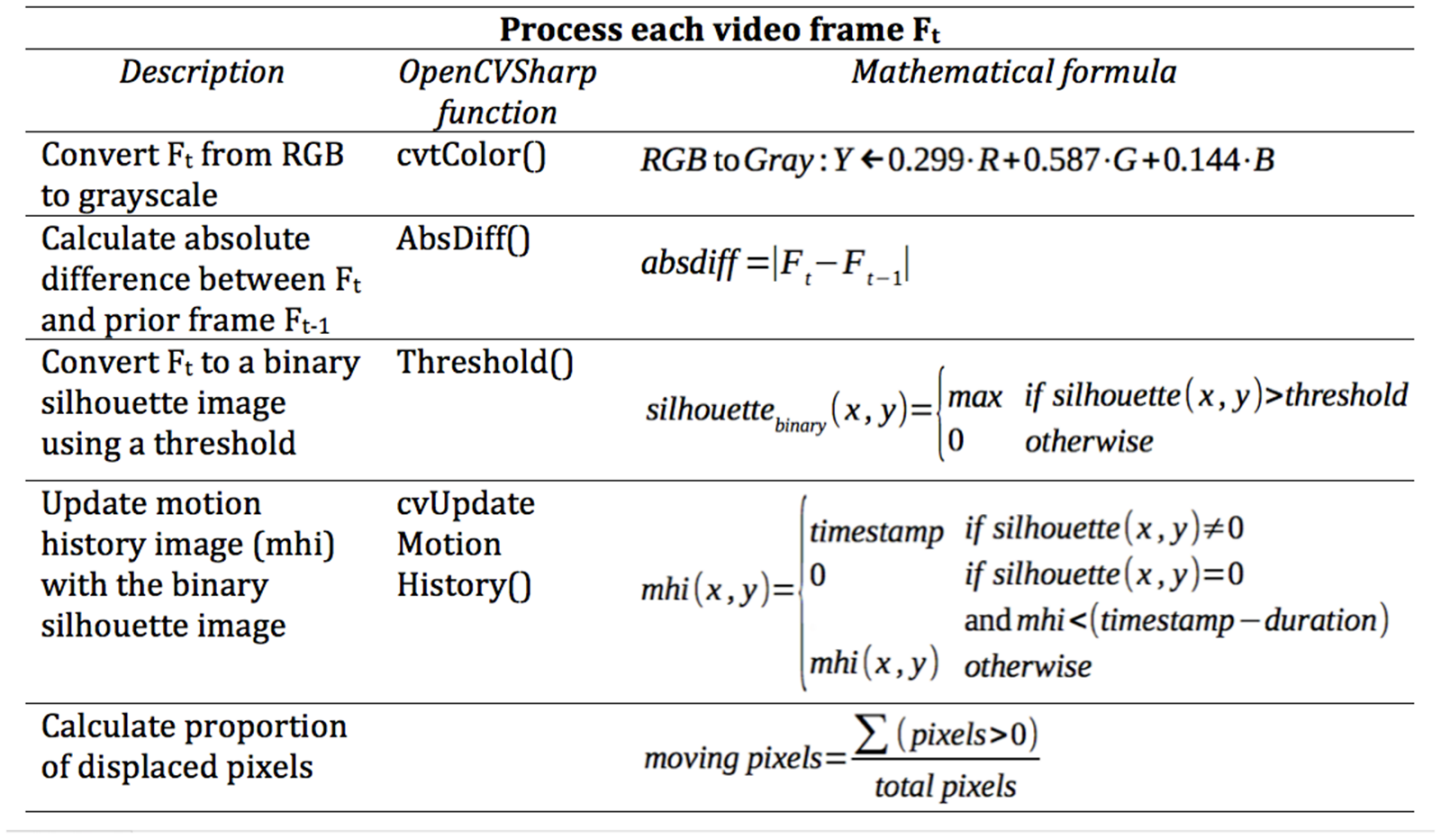
A flow chart depicting the steps taken when processing each frame of the video. The left column contains a description of each step, while the center column lists the corresponding OpenCvSharp function, and the right column shows the mathematical formula applied during that step.

Each video frame *F*_*t*_ is processed in three steps. The mathematical details of this processing are given in Fig 1, alongside the OpenCV functions applied (OpenCV is a library of programming functions for real time computer vision first introduced in [77]). First, *F*_*t*_ is converted to grayscale. Then the difference between *F*_*t*_ and the previous frame *F*_*t-1*_ is calculated to give a "silhouette" of the motion that has occurred. Third, the frame is thresholded to get a binary silhouette that has foreground pixels at maximal brightness and background pixels at minimal brightness. This is intended to capture the relevant foreground motion and filter out background noise [76,78]. The MHI is updated with the binary silhouette image as described above, giving us a composite motion image *M*_*t*_ that shows the most recent motion at maximal brightness. *M*_*t*_ is used to calculate the proportion of pixels in *F*_*t*_ that have been displaced, i.e., the index of the amount of movement in the frame.

Two main parameters in the algorithm can be adjusted to adapt the system to particular motion tracking scenarios: (a) *N*, the length of the MHI memory in milliseconds, and (b) *T*, the binary threshold value. The length of the MHI memory determines for how long frames are taken into account when determining current pixel intensity. Frames with timestamps older than the current time minus *N* are removed. If *N* is large, frames from a longer period of time are used to build the model and the system may not be as sensitive to small changes in motion (momentum-based smoothing). Ahad et al. [76] note that if *N* is too large, changes in pixel values are less significant. Vice versa, if *N* is small and fewer frames are used, small random changes in lighting may be mistaken for actual movements. In addition, *N* should not be shorter than the actions being studied, because then information about the actions is lost, decaying out of the memory before the actions are complete [76].

The binary threshold value determines how much background noise, such as noise from lighting changes, is filtered out. If *T* is too low, noise may not be filtered out as well. If *T* is too high, relevant features whose motion should be tracked may get lost. For further detail on setting a binary threshold, a well-studied problem in computer vision and image processing, see [79,80].

In our case, we tested multiple values with a small subset of our data, and through manual inspection of the results, found that the values *N* = 1000 ms and *T* = 30 gave acceptable results when tracking motion of the human torso and face or of the full body with the present approach, online and offline. We adopted this heuristic-based approach rather than a systematic parameter search to avoid prescribing parameters that overfit. This *N* is reasonable (following the advice of [76] on setting these parameters) given that we are considering changes in gross body movements, which occur on the scale of tens or hundreds of milliseconds, and thus would be recognized within the 1000ms memory window. It should be noted that since this algorithm may be applied offline, it is not necessary to set these parameters before collecting data. Instead, the parameters may be adjusted offline for various analyses that emphasize different timescales of movement.

### The Software

We implemented the motion tracking algorithm for Windows 7 machines using. NET using the OpenCvSharp library (a C# wrapper for OpenCV) (the OpenCVSharp library is available from https://github.com/shimat/opencvsharp/ and OpenCV is available from http://opencv.org/). OpenCvSharp includes functions for generating image silhouettes and for updating a MHI as described above. It also provides example code that was repurposed in our implementation. Currently, the software only runs on the Windows Operating System and has only been tested on Windows 7, although OpenCvSharp is inherently cross-platform. It should be noted that this algorithm could readily be ported to other platforms other than Windows, in other programming languages. For example, it could most easily be used with Mono (available from http://www.mono-project.com/), which is a cross-platform, open source. NET development framework that is binary compatible with Microsoft’s.NET platform. Other wrappers for OpenCV are also available, such as the EmguCV library (available from http://www.emgu.com/), which is a cross-platform. NET wrapper that can easily be compiled with Mono.

**Fig 2:**
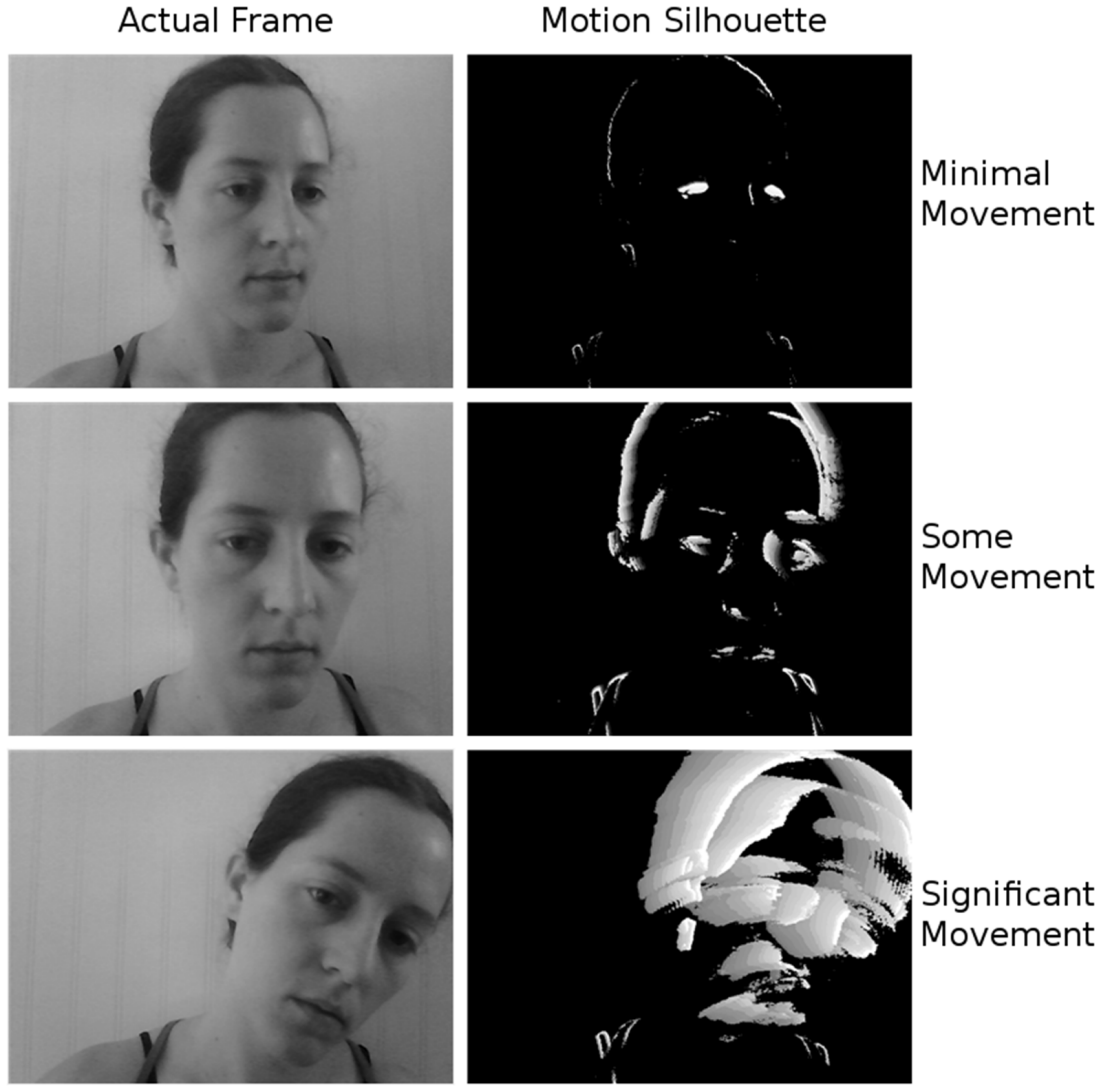
The Motion Tracker software interface in action. The video being processed is displayed in the top left panel, while the corresponding motion silhouette is shown at the top right. The lower left panel displays a moving graph of the motion index over time. Controls for the software, which allow the user to select which video to process, whether to show the visualizations, and where to save output files, are located in the lower right panel.

The Motion Tracker software that we developed provides a graphic user interface (GUI) for processing either pre-recorded or live video. Fig 2 displays a screen shot of the software. The GUI has four panels: 1) the current video frame (top left), 2) the corresponding motion silhouette (top right), 3) a moving time series of estimated movement (proportion of displaced pixels in each frame), and 4) controls for the software. Options are available to show or hide the visualizations and to set the internal parameters. Individual video files (e.g.,. avi,. mp4) as well as entire directories containing video files can be batch processed. The software generates a text file containing estimated movement for each frame in the video and the frame number. The software and an installation manual are available for download from http://jakory.com/motion-tracker/.

**Fig 3:**
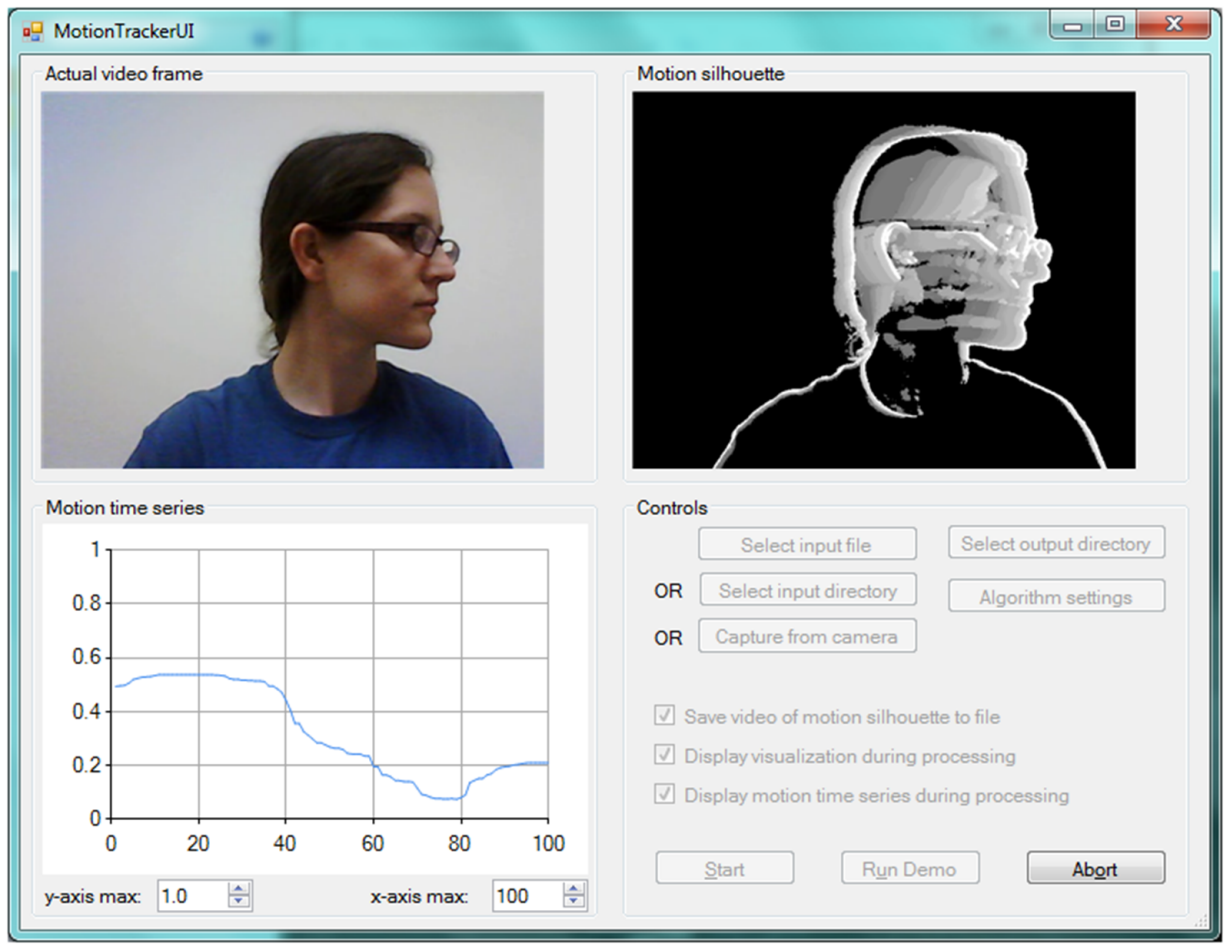
Sample output of the motion tracking algorithm. On the left are single frames extracted from a video sequence, while the panels on the right display the corresponding motion silhouettes. Pixels that have been displaced (i.e., places in the video frame where motion has occurred) are shown in white; pixels that have not been displaced are shown in black.

Fig 3 displays sample output of the motion tracking algorithm. The panels on the left show single frames taken from a video sequence, while panels on the right display the corresponding motion silhouettes. Pixels that have been displaced (i.e., places in the video frame where motion has occurred) are shown in white, while pixels that have not been displaced are shown in black. In the bottom panel, there is significant movement in the face and body. In contrast, in the middle panel, only some motion has occurred, and in the top panel, the body is motionless except for the eyes. As can be seen, the algorithm correctly filters out background noise such as static pixels and light fluctuation, and detects both small movement such as eye blinks when the head is still (top panel), intermediate movement such as slight shifts in posture (middle panel), and significant movements such as head tilts and nods (bottom panel).

## Validation Study 1

The first validation study consisted of analyzing data from an existing study that simultaneously recorded videos of participants’ faces and upper bodies as well as high-resolution maps of seated posture using the Body Pressure Measurement System [69]. As such, the subsequent sections focus on selective aspects of the previous study that are most relevant to the present study. Data used in this study are available for download at http://dx.doi.org/10.6084/m9.figshare.1425135.

### Participants

The participants were 28 college students from a Southern university in the United States. There were 5 males and 23 females. 37% of the students were white, 56% African American, and 7% were classified as “Other”. The participants received course credit for their participation. Data from one participant was discarded due to equipment failure. Written consent was obtained from all participants.

### Materials

#### Video

The video of the participant's upper body and face was captured using the IBM blue-eyes camera [81]. Although this is a specialized camera that can detect movements of the retina, eye blinks, and facial movement, we did not use these features; we simply recorded a 640x480 black-and-white video at 30 frames per second. The Motion Tracker software is expected to work equally well with video recorded by any webcam and with color-video provided that the lux—the total "amount" of visible light present—of the camera matches the illumination of the environment as demonstrated in Fig 2.

#### Body Pressure Measurement System (BPMS)

**Fig 4:**
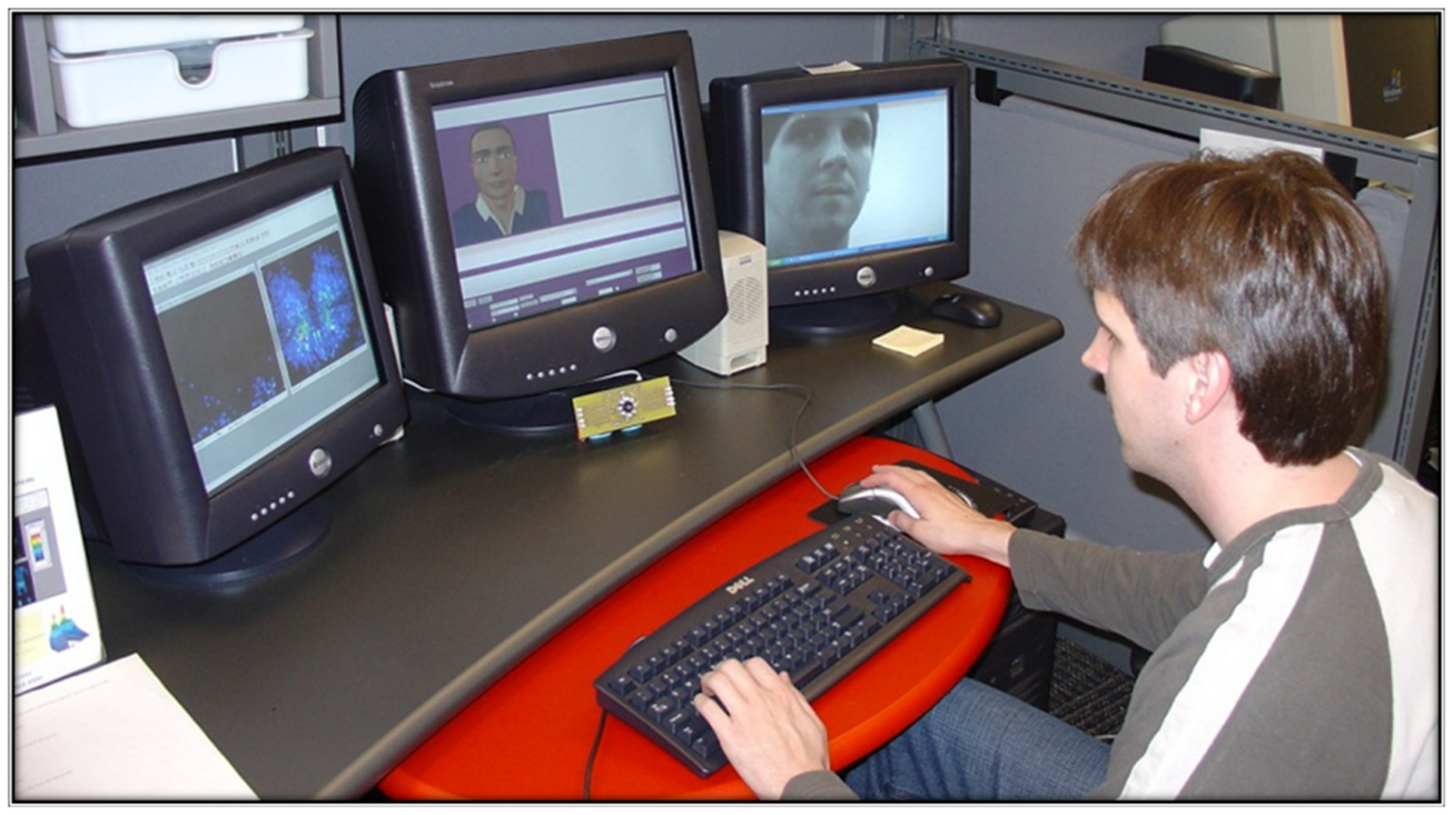
The overall experimental setup for the first validation study. The participant sat on a chair with the BPMS seat and pack pads, facing a computer monitor. The camera recorded the participant’s upper torso and face.

General movement was captured by the Tekscan BPMS [35], which consists of a thin-film pressure pad that can be mounted on different surfaces. The pad has a paper-thin rectangular grid of sensing elements enclosed in a protective pouch. Each sensing element provides 8-bit pressure output in mmHg. Our setup used two sensing pads, placed on the seat and back of a Steelcase Leap Chair. Data were recorded at a 4hz rate. Fig 4 shows the overall experimental setup.

### Procedure

Participants interacted with a computer tutor (AutoTutor, see [82]) for 32 minutes. Participants were told to take a comfortable, seated position and to feel free to move normally. During the interaction, data were recorded from the camera and the BPMS. All methods were approved by the University’s Institutional Review Board (IRB) prior to data collection. The individuals depicted in the images in this manuscript have given written informed consent (as outlined in PLOS consent form) to publish these case details.

### Data Preprocessing

#### Video

The videos recorded during the interactions were saved as compressed AVI files. Each of these videos was provided as input to our software, which computed a time series of the proportion of moving pixels on a frame-by-frame basis. We removed the first 90 data points (3 seconds) of each time series because it takes some time for a stable background model to be constructed. The exact number of data points to discard can be determined through manual inspection of the data—the first few values are generally much higher than all those following and can be identified as outliers (e.g., more than 2.5 standard deviations above the mean). The difference in the number of points excluded in this study versus in our second and third validation studies may be due to particular qualities of the cameras (different cameras were used in each study) or of the recording environment. For example, in this and in the third study, the start of each video corresponds to the start of video recording, while in the second study each video clip was cut from a longer video. If the camera takes a second or two to stabilize or adjust brightness at the start of a recording, this may correspond to the need to remove a greater number of data points in this and in the third study than in the second study.

Next, outliers were identified as z-scores more than 2.5 standard deviations from the mean and were removed from each time series. On average, 2.06% (*SD* = 1.85%, min = 0.15%, max = 5.59%) of the values were identified as outliers and were treated as missing values. To estimate *the magnitude of movement* (called estimated movement), we computed the absolute difference of the time series. For elements {x_1_, x_2_, x_3_ … x_n_} in the time series, the absolute difference of the series would be {|(x_2_—x_1_)|, |(x_3_—x_2_)| … |(x_n-_x_n-1_)|}. This is the primary measure extracted from the videos and used in all subsequent analyses. We used this measure because the transformation to a difference time series makes the motion estimated from the videos and the motion from the BPMS comparable.

**Fig 5:**
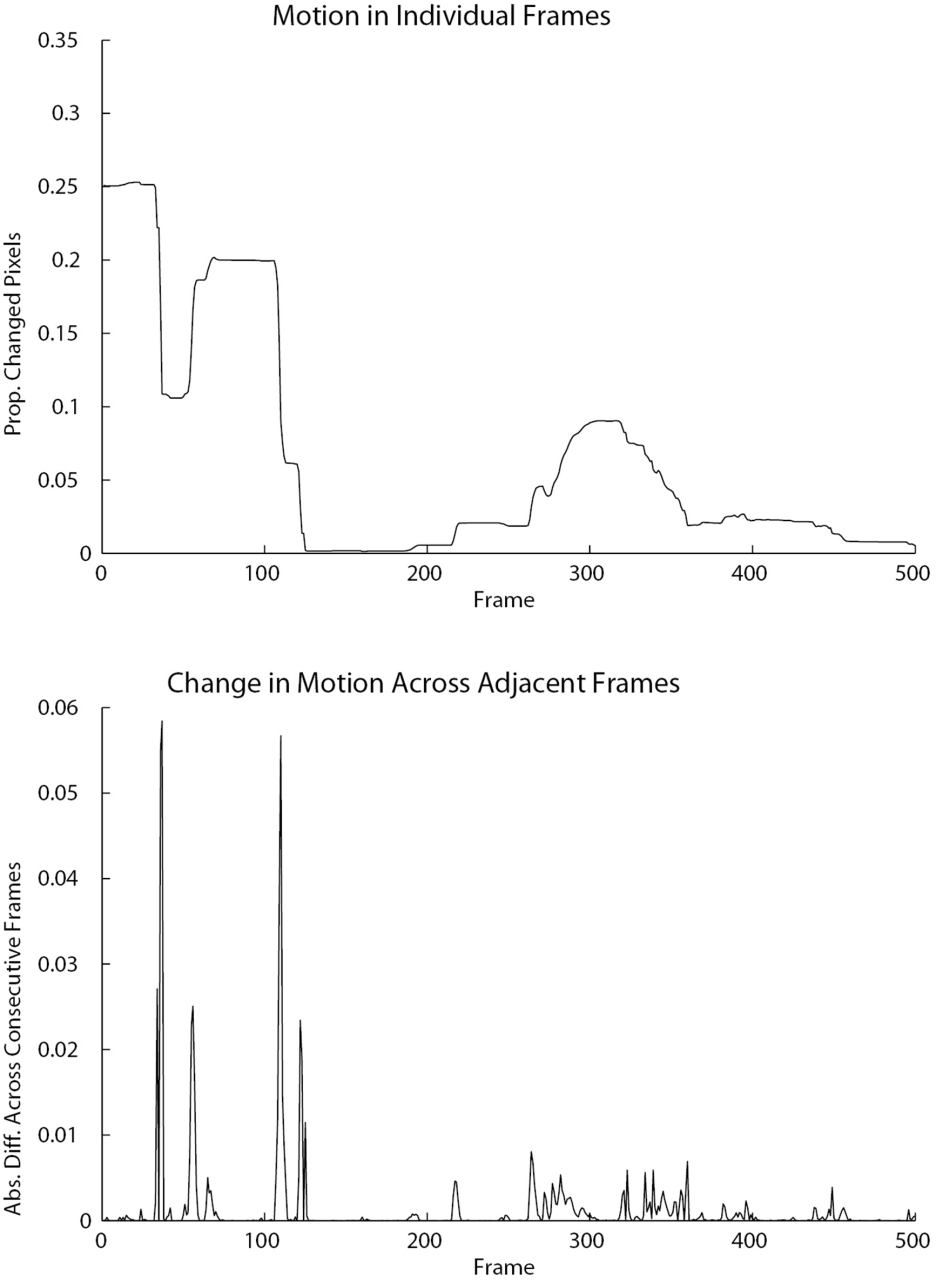
These graphs display a 500 timestep segment of a sample motion time series. The top graph shows motion in individual frames as the proportion of changed pixels per frame, while the bottom graph shows the absolute difference of the proportion of changed pixels per frame across consecutive frames, i.e., the change in motion across adjacent frames. Periods of stable motion in the top graph are reflected by small spikes in the absolute difference graph, i.e., small changes in motion across adjacent frames. Sharp increases or decreases in motion are reflected by larger spikes, indicative of larger changes in motion across adjacent frames.

Fig 5 displays a 500 timestep segment of a participant’s time series. Recall the video was recorded at 30 frames per second. The top graph displays motion in the individual frames as the proportion of changed pixels per frame. The bottom graph displays estimated movement, which is the absolute difference of the proportion of changed pixels across adjacent frames. Periods of stable motion in the top graph are reflected by small spikes in the bottom graph. The small spikes are indicative of small changes in movement across adjacent frames. Larger spikes in the bottom graph indicate larger changes in movement across adjacent frames, reflecting sharp increases or decreases in the amount of movement. For example, in the top graph there is stable movement until approximately frame 30, at which point there is a sharp decrease in the amount of movement. This is reflected in the bottom graph as a series of very small spikes during the period of stable movement, followed by a large spike when the movement suddenly decreases.

#### BPMS

The output from the BPMS is a 38 × 41 pressure matrix (rows x columns) for each pad, with each cell in the matrix corresponding to the amount of pressure exerted on a single sensing element. The key dependent variable computed for both the back and seat pads was the mean pressure exerted on the pad in each frame. It was computed by summing the pressure exerted on each sensing element in the pad and dividing the sum by the total number of elements. The result was two time series, one for the back and one for the seat. As with the motion time series, we removed the first 12 steps (3 seconds because BPMS is sampled at 4 Hz) of each back and seat time series. We then computed the absolute difference of each time series, using the same method as described above.

**Fig 6:**
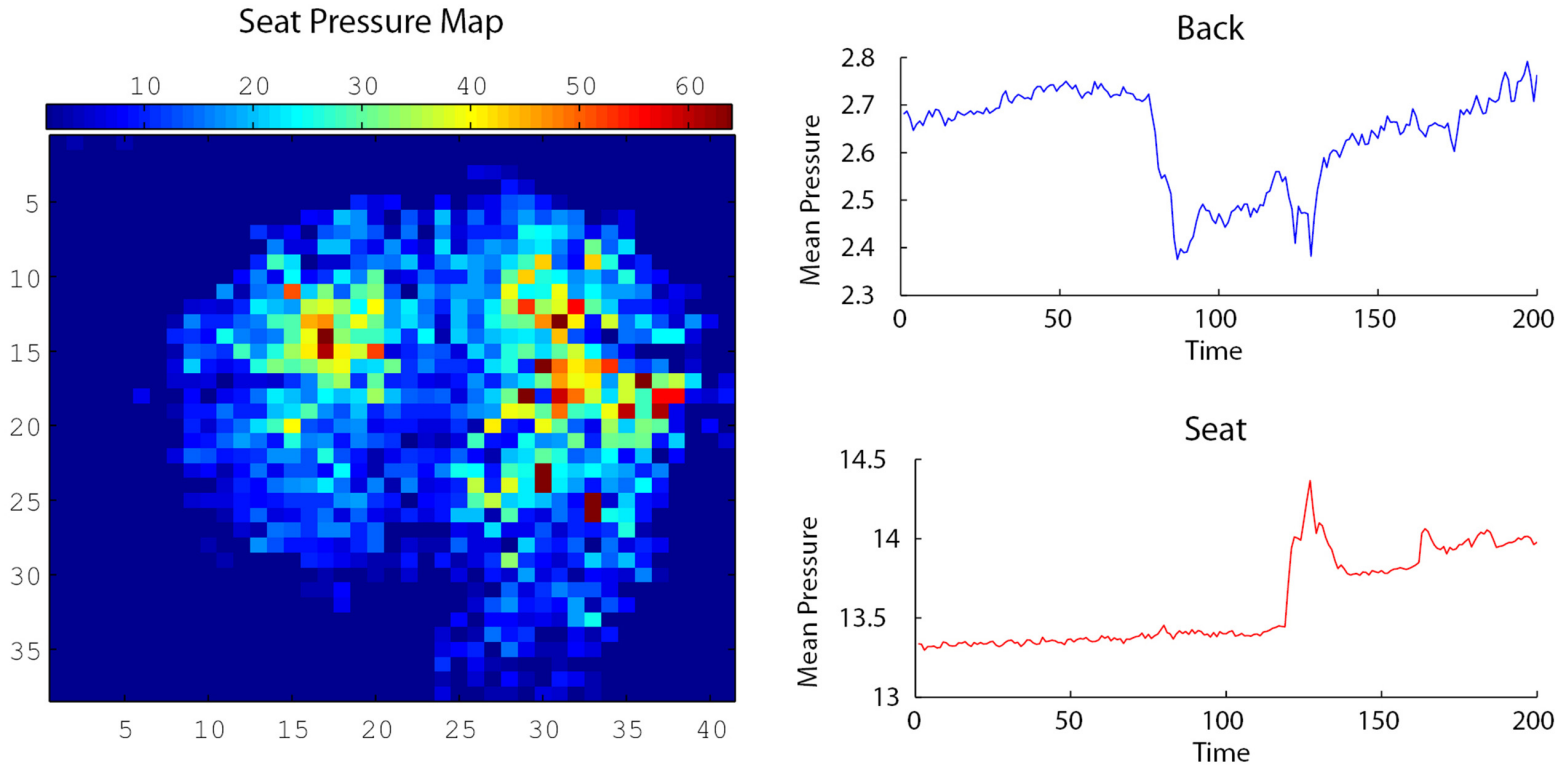
Sample output from the BPMS pressure pads. On the left is a pressure map from the seat pressure pad. Each square in the map corresponds to a single sensing element. On the right are graphs showing a 200 timestep segment of a sample mean pressure time series for the back pressure pad (top) and for the seat pressure pad (bottom). Changes in pressure against the seat and back pads are reflected in the spikes and dips in the mean pressure graphs.

Fig 6 shows sample output from the BPMS pressure pads. The left image shows a pressure map from the seat pad. Each square in the map corresponds to a single sensing element. On the right are graphs showing a 200 segment of a sample mean pressure time series for the back pressure pad (top) and for the seat pressure pad (bottom). Changes in pressure on the back pad or on the seat pad are reflected in the spikes and dips in the mean pressure graph. These were subsequently captured in the absolute difference time series (not shown here). For example, in the back time series, pressure is initially fairly stable, but there is a drop in mean pressure at about 85 time steps, which might be indicative of a forward lean.

#### Analysis

The data relevant to the present analyses consist of absolute difference time series of (1) the motion estimated from the face and upper body (called estimated movement), (2) the motion from the BPMS seat pad (called seat movement), and (3) the motion from the BPMS back pad (called back movement). Each participant contributed one of each of these three types of time series, thereby yielding 81 time series in all (3 time series × 27 participants). Data were analyzed at two levels, across participants and between participants. First, we calculated the mean for each time series and computed bivariate correlations among these three means. This analysis focuses on how these various time series correlate *across* participants. Second, we divided each time series into 10 windows of equal width, computed the mean of each window, created time series of these windowed means, and computed cross-correlations among the three windowed time series. This second analysis investigates correlations *within* a participant’s session.

### Results and Discussion

#### Correlations across participants

**Fig 7:**
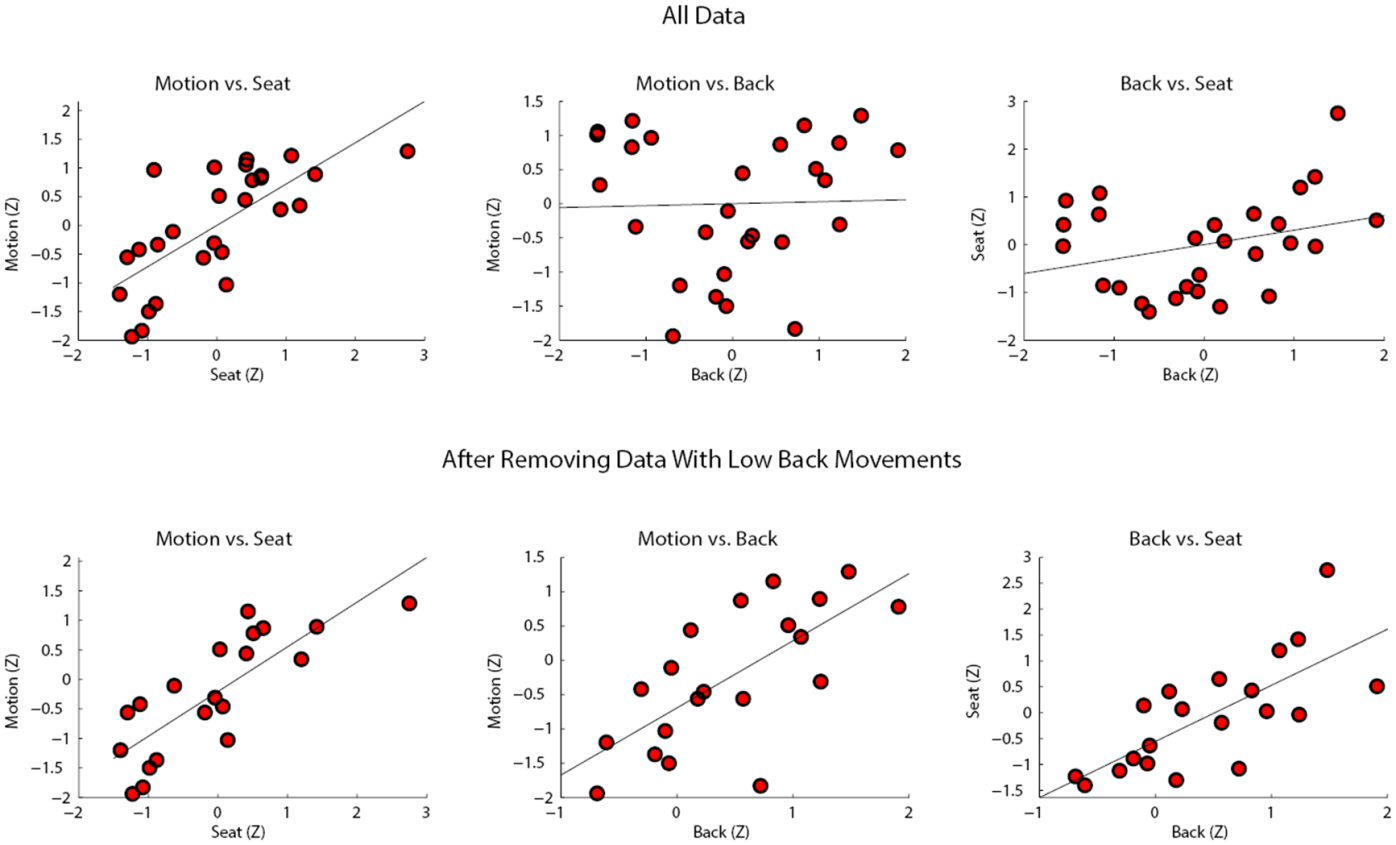
Scatter plots showing the mean of the absolute difference of each time series (as z-scores) vs. each other time series. The top panel includes all data while the bottom panel eliminates participants with negligible back movement.

As can be seen in the top panel of scatter plots in Fig 7, estimated movement was significantly positively correlated with the seat movement (*r* = .720, *p* < .001), but was not significantly correlated with back movement (*r* = .029, *p* = .887). Seat and back movement were weakly correlated (*r* = .303, *p* = .125). The low correlation between seat and back movement from the same sensor was surprising, so we examined the data more closely. An examination of the scatter plots in Fig 7 indicated that the lack of a substantial correlation between the back and the other time series can be attributed to a subset of participants with very little movement in the back (see cluster of points on the top left of the middle and right plots in the top panel of Fig 7). We identified these points as participants whose mean back values were below the 25^th^ percentile. After eliminating these seven data points, back movement now reliably correlated strongly with estimated movement (*r* = .695, *p* = .001) and with seat movement (*r* = .727, *p* < .001) (see bottom panel of Fig 7). Seat movement and estimated movement were still significantly correlated (*r* = .804, *p* < .001). The correlations are presented in Table 1.

**Table 1:** Means and standard deviations (in parentheses) of cross-correlations among windowed time series.

Data Type	Lag 0	Best of Lags -1, 0, 1
**All Data**		
Motion & Back	.382 (.410)	.507 (.301)
Motion & Seat	.562 (.213)	.606 (.202)
Back & Seat	.661 (.324)	.687 (.284)
**Subset of Data**		
Motion & Back	.426 (.420)	.525 (.232)
Motion & Seat	.527 (.288)	.756 (.236)
Back & Seat	.757 (.232)	.757 (.232)

These results suggest that back movement as measured with the BPMS does indeed correlate with estimated movement, when movement is actually being measured by the BPMS back pad. An examination of the videos revealed that participants with minimal back movement were leaning forward during the majority of the recording session. Because their backs were not against the BPMS back pad, no reliable data about their back movements were recorded. As such, because the BPMS back pad was not measuring their body motion, that data should be discarded. An independent samples t-test indicated that the mean seat values of participants with minimal back movement (*M* = .037, *SD* = .011) did not significantly differ from those with higher back movement (*M* = .033, *SD* = .015), *t*(25) = .544, *p* = .591. This suggests that movement did occur, but was simply not detected by the back pad because there was no pressure on the back. Removal of these participants is justified for the present analysis because the problem lies with the back sensor and not with our vision-based system that reliably correlated with the seat sensor. As discussed below, our second validation study demonstrates that the Motion Tracker correlates well with a different sensor worn by people performing a different task, suggesting that the irregularities seen here were due to the back sensor in this particular task.

#### Cross correlations within participants

We examined the cross-correlations to see how the windowed estimated movement time series correlated with the windowed seat and back movement time series. Cross correlation coefficients were separately computed for each participant at lags -1, 0, and 1. However, we initially examined just the lag zero cross correlation, because we expected that, since the time series were synced in time, the strongest correlation would at lag zero. The formula for computing a normalized cross correlation C between two signals, *x* and *y* of length *n*, with time shift or lag *k* is as follows [83]:

$$C_{xy}\left(k\right)\equiv \{\begin{array}{c} \frac{1}{n}\overset{n-k}{\underset{t=1}{\sum }}(x_{n}-\bar{x})\left(y_{n+k}-\bar{y}\right),\;k=0,\;1,\;2,\ldots \\ \frac{1}{n}\overset{n-k}{\underset{t=1}{\sum }}(x_{n}-\bar{x})\left(y_{n+k}-\bar{y}\right),\;k=-1,\;-2,\ldots \end{array}$$

**Fig 8:**
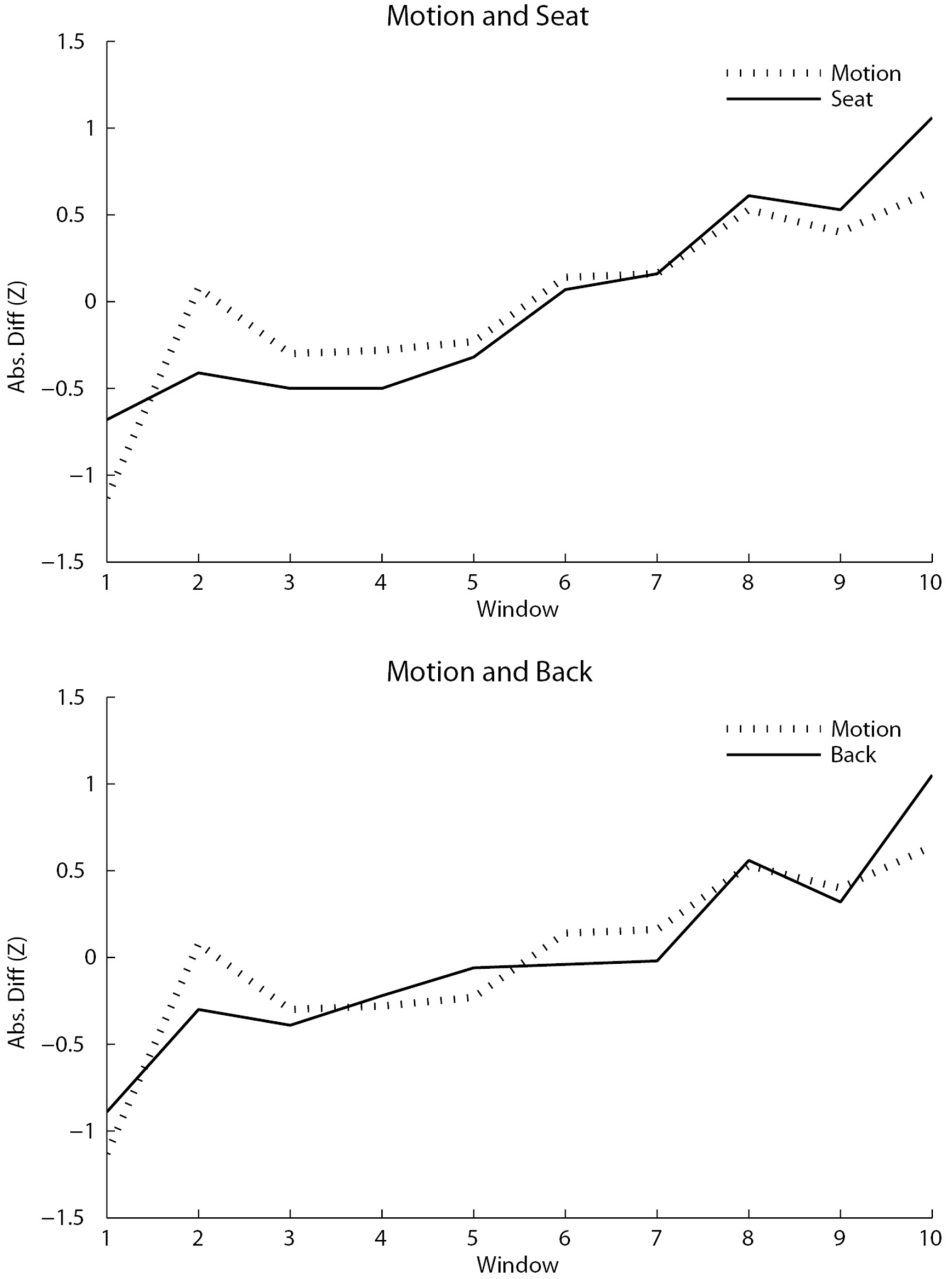
Line graphs of the mean cross-correlation across all participants of the mean of the absolute difference of each time series (z-scores), with each time series divided into 10 windows. The top graph shows the motion and seat over time, while the bottom graph shows the motion and back over time.

Means and standard deviations of the cross correlations (across participants) were then computed for each lag. The lag zero mean (across participants) cross correlation between estimated movement and seat movement was significantly greater than zero (*M* = .562, *SD* = .213, *t*(26) = 13.7, *p* < .001, *d* = 2.13 σ) as was the cross correlation between estimated movement and back movement, (*M* = .382, *SD* = .410, *t*(26) = 4.83, *p* < .001, *d* = 0.932 σ for motion and back). Lag zero cross correlations between windowed-time series for back and seat movement were also significantly greater than zero (*M* = .661, *SD* = .324, *t*(26) = 10.6, *p* < .001, *d* = 2.04 σ). Fig 8 shows graphs of the mean cross correlations involving the estimated movement at lag zero. Removing participants with low back movements improved the cross-correlations involving the back (see Table 1).

There was the possibility that for some participants the strongest correlation between the windowed time series was in fact at lag -1 or lag 1, rather than at lag 0. This would occur when the time series are not perfectly synced in time, with one series lagging behind the other. We found that if we considered the strongest positive correlation among lag -1, 0, or 1, then in general, the cross-correlation was much stronger (mean improvement 18.5%, min 0%, max 43.4%) and had less variance (mean decrease in variance 17.8%, min 0%, max 44.8%), as listed in Table 2. The lag values of seven participants were changed by this consideration. This may be due to an initial temporal misalignment in these participants' BPMS and video recordings. Our third validation study addresses this question by showing that in a directed task, with careful alignment of the data streams, no irregularity in the cross-correlation is found.

**Table 2:** Means and standard deviations (in parentheses) of cross-correlations among windowed time series.

Data Type	Lag 0	Best of Lags -1, 0, 1
**All Data**		
Motion & Back	.382 (.410)	.507 (.301)
Motion & Seat	.562 (.213)	.606 (.202)
Back & Seat	.661 (.324)	.687 (.284)
**Subset of Data**		
Motion & Back	.426 (.420)	.525 (.232)
Motion & Seat	.527 (.288)	.756 (.236)
Back & Seat	.757 (.232)	.757 (.232)

## Validation Study 2

The goals of the second validation study were twofold: (1) to demonstrate that the Motion Tracker correlates with a second, different contact-based sensor, in this case an accelerometer, and (2) to determine whether the irregularities in the first study’s correlations regarding participants with low back movement were a result of the back sensor, not due to a failure of the Motion Tracker itself. To these ends, the second validation study consisted of analyzing data from the University of Texas at Dallas Multimodal Action Dataset (UTD-MAD) [70]. The UTD-MAD includes recordings from a Kinect and a wearable inertial measurement unit. Here we analyzed the video of participants’ full bodies and data from three-axis accelerometer worn on the right wrist. This data is freely available for download from http://www.utdallas.edu/~cxc123730/UTD-MHAD.html. Below, we briefly describe the participants and relevant materials. This information comes from [70]; we refer the reader to that paper and the website linked above for further details about the UTD-MAD.

### Participants

The participants were 8 university students. There were 4 males and 4 females. Additional information about the participants was not available from the dataset.

### Materials

#### Video

The video of the front of the participant’s full body was captured with a video camera at 30 frames per second with a resolution of 640x480 pixels.

#### Accelerometer

One wearable inertial sensor built at the ESSP Laboratory at the University of Texas at Dallas was worn on the participant’s right wrist or, for some actions consisting primarily of leg movement, on the right thigh. The sensor included a 3-axis accelerometer that recorded motion data at 50 Hz.

### Procedure

Participants were recorded while performing four repetitions of each of twenty-seven actions, such as waving, throwing, pushing, walking, and jogging in place. Each action was only a few seconds long. Since many of these actions involved both arms or a great deal of body movement that would not be picked up by a single wrist or thigh-worn sensor, we selected the top three actions whose motion could be sufficiently captured by just that one sensor. These actions were (1) right arm swipe to the left, (2) right arm swipe to the right, and (3) right hand wave. Thus, each participant contributed 12 time series (3 actions x 4 repetitions), for a total of 84 action time series (7 participants x 3 actions x 4 repetitions).

### Data Preprocessing

#### Video

We followed the same procedure as in the first validation study: Each video was provided as input to our software, which computed the time series of the proportion of moving pixels. The first six frames (1/5 second, because the video was recorded at 30 Hz) of each time series were removed to allow a stable background model time to be constructed. Outliers identified as z-scores more than 2.5 standard deviations from the mean were removed. On average, 0.97% (*SD* = 1.12%, min = 0.00%, max = 4.44%) of the values were identified as outliers and treated as missing values. Then, as we did during the first validation study, we computed the absolute difference of the time series. We used the exact same parameters as Study 1 in order to test generalizability of the parameter set. We also did not separately estimate parameters for each action as this might amount to overfitting.

#### Accelerometer

The accelerometer provided a time series of data in each of the x, y, and z dimensions. From these three dimensions, we computed a single time series for the accelerometer consisting of the Euclidean norm of the x, y, and z series at each time point. The Euclidean norm is the square root of x^2^ + y^2^ + z^2^. Then, as with the motion series, we first removed the first ten steps (1/5 second, because the accelerometer was recorded at 50 Hz). We excluded one participant from further analysis because their acceleration data was on average more than 2 standard deviations from the mean for all three actions. Two repetitions of action 3 from another participant were similarly removed. We did not compute the absolute difference of the time series because the accelerometer already measures changes in velocity, which is comparable to what the absolute difference of the estimated motion time series measures.

#### Analysis

The analysis followed the same procedure as during the first validation study. The data relevant to the analysis consisted of (1) absolute time series of the motion estimated from the full body (estimated movement), and (2) the norm time series of motion from the wrist accelerometer. Each participant contributed one of each type of time series for each repetition of each action, yielding 164 time series in total (2 time series x 7 participants x 3 actions x 4 repetitions, minus the two excluded repetitions). Then we calculated the mean for each time series and computed bivariate correlations among these means. We did not compute cross-correlations because the time series were too short for the cross-correlations to be meaningful—each recorded action was only one or two seconds long.

### Results and Discussion

**Fig 9:**
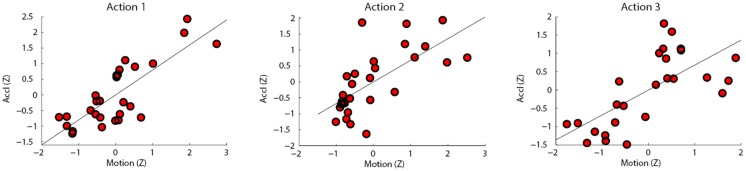
Scatter plots showing the mean of the absolute difference of the estimated movement vs. the accelerometer time series (as z-scores), for each of the three actions performed by subjects: (1) right arm swipe to the left, (2) right arm swipe to the right, and (3) right hand wave.

The estimated movement was significantly positively correlated with the accelerometer movement for all three actions (action 1: *r* = .801, *p* < .001; action 2: *r* = .679, *p* < .001; action 3: *r* = .681, *p* < .001), as shown in Fig 9. These results suggest that the irregularities found in Study 1, such as the low correlations for participants who exhibited low back movement, were due to the nature of the sensor in Study 1, not due to a failure of the Motion Tracker itself.

## Validation Study 3

In the first study, we saw that for some participants, the cross-correlation between movement data from the Motion Tracker and the BPMS as stronger at lag 1 or lag -1 than at lag zero, possibly because the data recordings were misaligned. The purpose of this third validation study was to demonstrate that in a directed task, with careful alignment of the data streams, the cross-correlation between movement data from the Motion Tracker and from another system is strongest at lag zero and not irregular—i.e., that the irregularities seen in the first study were a result of that study’s data recordings, not a result of the Motion Tracker. In this study, we analyzed a 5-minute video of a person seated at a desk and corresponding data recorded with a Kinect of the person’s head pose. Data used in this study are available for download at http://dx.doi.org/10.6084/m9.figshare.1425135.

### Participants

One male was recorded.

### Materials

#### Video

The video of the front of the participant’s body while seated at a desk was captured with the Kinect’s RGB video camera at 30 frames per second with a resolution of 640x480 pixels.

#### Kinect skeleton

The Kinect was used to record the position of the participant’s head at 30 Hz. We used the head tracking position, as opposed to other specific joints, because given the task, it seemed most comparable to the gross body movement measured by the Motion Tracker.

### Procedure

The participant was instructed to sit without leaning on the desk or leaning back against the chair for five minutes.

### Data Preprocessing

#### Video

We followed the same procedure as in the first validation study and used the same parameters. The first thirty frames (1 second, because the video was recorded at 30 Hz) of the time series were removed to allow a stable background model time to be constructed. Outliers identified as z-scores more than 2.5 standard deviations from the mean were removed; 0.5% of the values were identified as outliers and treated as missing values. Then, as we did during the first validation study, we computed the absolute difference of the time series.

#### Kinect

The Kinect provided a time series of the x, y, and z position of the participant’s head. From these three dimensions, we computed a single time series consisting of the Euclidean norm of the x, y, and z series at each time point. Then, as with the motion series, we removed the first thirty steps (1 second at 30 Hz). We then computed the absolute difference of each time series.

#### Analysis

The analysis followed the same procedure as during the first validation study. The data included one absolute difference time series of each of (1) the motion estimated from the face and upper body, and (2) the motion from the Kinect skeleton centroid. Because there was only one participant, this analysis focused on how the time series correlated *within* the participant’s session. As we did in the first study, we divided each time series into 10 windows of equal width, computed the mean of each window, created time series of these windowed means, and computed cross-correlations among the two windowed time series.

### Results and Discussion

**Fig 10:**
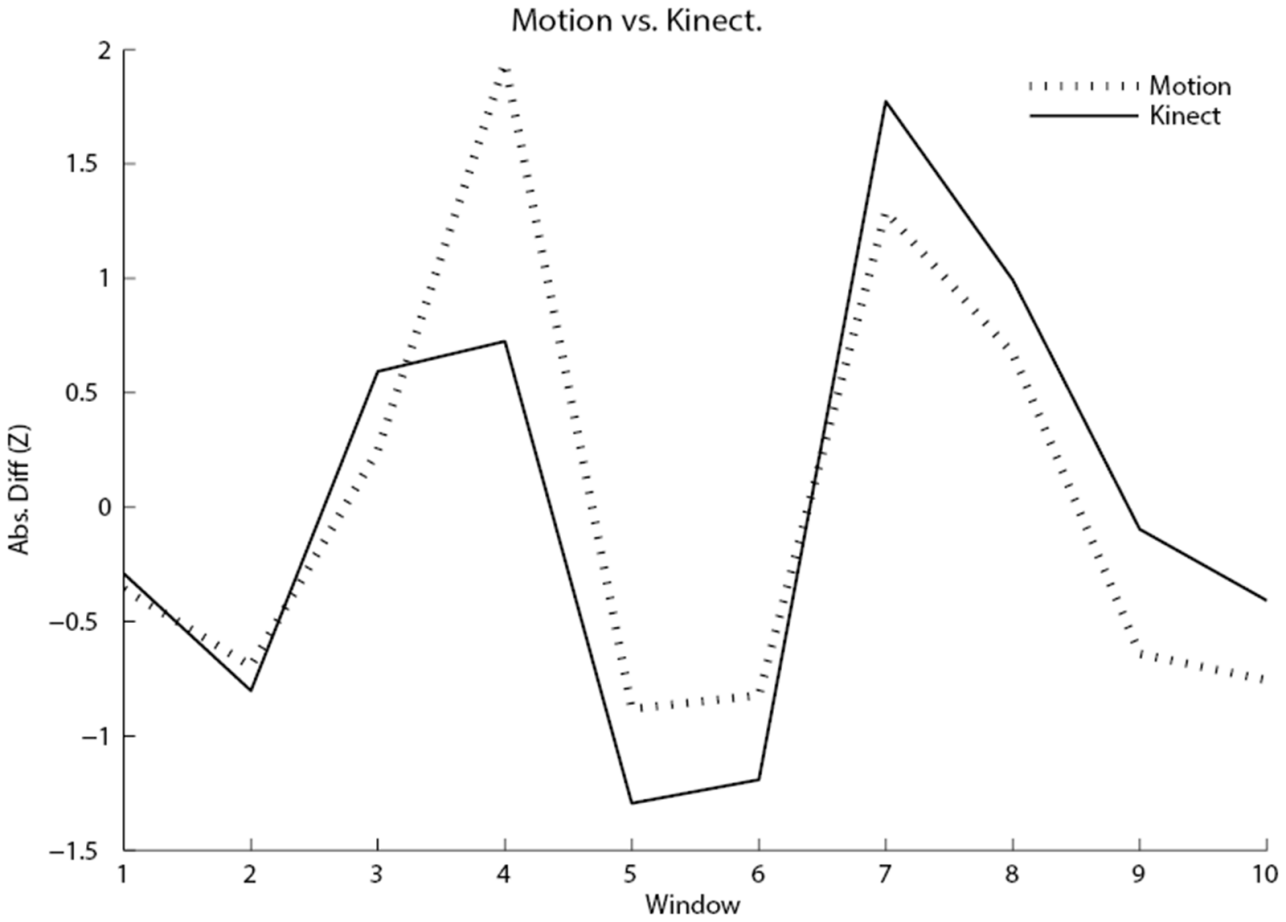
Line graph of the cross-correlation of the absolute difference of the estimated movement and the Kinect head position time series (z-scores), with each time series divided into 10 windows.

As in the first study, cross correlation coefficients were separately computed at lags -1, 0, and 1. However, in this case, the lag zero cross correlation between estimated movement and the Kinect movement was by far the strongest (lag 0 = .855, lag -1 = -.135, lag 1 = .104). Fig 10 shows a graph of the cross correlation involving the estimated movement at lag zero. This suggests that given proper alignment of data streams, estimated movement will correlate as expected with data from other movement sensors.

## General Discussion

We have demonstrated that the measure of movement obtained from the Motion Tracker software correlates strongly with (1) a proprietary body pressure measurement system (BPMS) (2) an accelerometer worn on the wrist, and (3) the Kinect’s head-tracking. This approach could be highly useful to researchers in small or low-budget labs, who have minimal programming experience or who want a plug-and-play system, while also allowing for simple and ecologically valid research designs. It is cost-effective because it does not require any customized hardware other than a simple web-camera. It is non-intrusive because it does not involve any wearable technologies. It is expected to be portable and can be deployed with relative ease, at least compared to some of the alternate technologies. In addition, its near real-time performance affords the possibility to not only record data in real-time, but also use the real-time data to modulate stimuli presented to the participant. This is useful for building responsive systems that adapt to users’ nonverbal behavior. For example, [84] used feedback about a person’s posture to improve the rapport-building behavior of a 3D conversational agent.

Finally, as we demonstrated in our validation studies, the software can be used to analyze bodily movement from existing data sets and large databases that include a video channel. For example, the SEMAINE database includes video recorded from frontal and profile views of people's faces and bodies from both grayscale and color cameras during a conversational interaction between a person and four emotionally stereotyped computer characters [85]. The interaction was designed to elicit emotional behavior from the participant. A collection of popular human motion databases was surveyed by Guerra-Filho and Biswas [86]. These include video of human actions, sequences, expressions, and emotions. The TalkBank and CHILDES databases contain video of conversations with adults and children, which may also be suitable (see [87,88]).

Some evidence for the system’s utility as an important tool for research in the cognitive and affective sciences can be found in one of our recent research studies [13]. This study focused on investigating how affective states of frustration, confusion, and anxiety modulate presumably unconscious subtle bodily movement. It used 1/f noise, pink noise, or “fractal scaling” as a measure of the low-level dynamics of body movement. This is an extremely sensitive measure that is notoriously difficult to appropriately compute because it is strongly influenced by extraneous noise in a time series [89]. Study 1 tracked 1/f noise in body movements that were monitored with the BPMS system while Study 2 used the Motion Tracker to estimate 1/f noise in movements of a different set of students. The major patterns discovered in Study 1 were replicated in Study 2 despite the sensitivity of the measure and the fact that both studies used different methods to track body movement.

### Limitations, Issues, and Recommendations

We have noted multiple limitations of the Motion Tracker software throughout this paper, which we summarize here. We also list some practical issues and recommendations pertaining to use of the Motion Tracker software. First, the software is at present written in C# and is Windows-only. The easiest way to run it on another operating system is likely through use of the open-source, cross-platform Mono Framework. The algorithm could also be ported to any other computer programming language, which would be relatively straightforward for any language with an existing OpenCV library (e.g., Python). Second, several parameters in the algorithm may have to be tuned for specific applications. We have provided the values of the parameters that we used, and the GUI makes further tuning quite easy. We used a heuristic-based approach to determine good parameter values, rather than a systematic parameter search, to avoid overfitting. The good performance of the Motion Tracker with these parameters on both datasets in the validation studies, despite the fact that the datasets were so different, is a testament that this approach worked well. Third, it may take several seconds for a stable background model to be built; as such, the first few values for estimated movement should be discarded (as we did during the validation studies). Exactly how long may depend on the specific data, but can be determined through manual inspection of the data—the first few values are often much higher than all those following and will be outliers (e.g., more than 2.5 standard deviations above the mean). The difference seen in the number of points excluded in each of our validation studies may be due to particular qualities of the different cameras, or the different recording environments.

This measure of movement is dependent on having a video of adequate quality. As with any computer vision based solution, extremely noisy backgrounds with rapidly changing lighting and multiple moving objects in the video can decrease the accuracy of the algorithm. For best results, we recommend using controlled lighting in a room with a relatively static background, with the camera focused on the participant alone. As noted earlier, we expect the software will work best when the lux—the total “amount” of light present—of the camera matches the illumination of the environment. Panning and zooming should be avoided to avoid confounding camera movement with participant movement.

In addition, the movement estimates will be more accurate if the entire body region of interest is within the video frame and if camera position is consistent across all participants. Indeed, if one is studying movement during activities that include walking or changing location, then it may not be practical to use a stationary camera that is tethered to a computer. Even with seated tasks, however, care must be taken to position the camera accordingly—for example, for participants of different heights. Many laptops and monitors now have integrated webcams that have been designed to capture only the head and shoulders of a user for videoconferencing, so recording of different body regions may require an external camera that can be re-positioned as needed.

We should also note that the present approach tracks only general body movements, not specific gestures or effectors. There is the concern that small movements, such as eye blinks, may not be registered well by the BPMS in the first validation study or by the accelerometer in the second validation study, but would be detected by our vision-based system. However, compared to motion from head tilts, leaning forward or backward, and other upper body movements, eye blinks generally comprise a relatively small portion of the total motion in any given frame. As such, the fact that they are registered by our motion tracker but may not be registered by the BPMS/accelerometer will have only a very small effect in the long run on the accuracy of the correlations we found. Finally, it is important to emphasize that the proposed system estimates one dimension of bodily movement from a video stream at a fraction of the cost of any proprietary system. The method is applicable for movements in two spatial dimensions (the x-y plane of the video); further work as described below is required to extend it to three. It is not intended to substitute for the high resolution spatial maps provided by the BPMS, the three-dimensional tracking of Vicon or Optotrak systems, and the level of detail that other systems provide.

That being said, we are in the process of increasing its effectiveness in a number of ways. In particular, the current study used the proportion of moving pixels in the *entire frame* as an index of movement. However, it is possible to segment the video into different spatial regions and independently estimate movement in each region. This would be particularly useful if one is interested in segregating upper and lower body movement or if there are two conversational partners in the frame and the goal is to track coordination in movement over time. Some existing work that segments video frames in order to track interpersonal synchrony includes [61,62,64]. It is also possible to use computer vision algorithms to detect the face [90] and estimate movement in the facial region alone. The distance of the face from the camera can then be estimated based on changes in the size of the detected face, thus extending the system into another spatial dimension. These and further refinements of the software await future research and technological development.
