# Correction: Motion Tracker: Camera-Based Monitoring of Bodily Movements Using Motion Silhouettes

**DOI:** 10.1371/journal.pone.0136481

**Published:** 2015-08-19

**Authors:** The PLOS ONE Staff

The first author’s name is improperly abbreviated in the citation. The publisher apologizes for this error. The correct citation is: Kory Westlund J, D’Mello SK, Olney AM (2015) Motion Tracker: Camera-Based Monitoring of Bodily Movements Using Motion Silhouettes. PLoS ONE 10(6): e0130293. doi:10.1371/journal.pone.0130293
