# Correction: Motion Tracker: Camera-Based Monitoring of Bodily Movements Using Motion Silhouettes

**DOI:** 10.1371/journal.pone.0138636

**Published:** 2015-09-17

**Authors:** Jacqueline Kory Westlund, Sidney K. D’Mello, Andrew M. Olney

Table 1 was erroneously published twice, once as Table 1 and once as Table 2. All references to Table 2 refer to Table 1.

The following information is missing from the Funding section: This research was supported by the National Science Foundation (NSF) (ITR 0325428, HCC 0834847, DRL 1235958, and Graduate Research Fellowship under Grant No. 1122374). Any opinions, findings, conclusions, or recommendations expressed are those of the author and do not reflect the views of the NSF. The funders had no role in study design, data collection and analysis, decision to publish, or preparation of the manuscript.
